# Sonic Kayaks: Environmental monitoring and experimental music by citizens

**DOI:** 10.1371/journal.pbio.2004044

**Published:** 2017-11-30

**Authors:** Amber G. F. Griffiths, Kirsty M. Kemp, Kaffe Matthews, Joanne K. Garrett, David J. Griffiths

**Affiliations:** 1 FoAM Kernow, Penryn, United Kingdom; 2 Institute of Zoology, Zoological Society of London, London, United Kingdom; 3 The Bicrophonic Research Institute, AudRey, London, United Kingdom; 4 European Centre for Environment and Human Health, University of Exeter, Knowledge Spa, Truro, United Kingdom

## Abstract

The Sonic Kayak is a musical instrument used to investigate nature and developed during open hacklab events. The kayaks are rigged with underwater environmental sensors, which allow paddlers to hear real-time water temperature sonifications and underwater sounds, generating live music from the marine world. Sensor data is also logged every second with location, time and date, which allows for fine-scale mapping of water temperatures and underwater noise that was previously unattainable using standard research equipment. The system can be used as a citizen science data collection device, research equipment for professional scientists, or a sound art installation in its own right.

## Introduction

The essence of scientific research is the exploration of the unknown and the discovery of something new. Likewise, understanding and explaining the world is one of the core foundations of the arts and music. New low-cost distributed manufacturing technology and the widespread sharing of protocols and hardware design through the internet mean the power to do scientific research no longer needs to be confined to expensive research labs.

With more accessible hardware comes the opportunity for more ambitious and adventurous science education and engagement that is inherently merged with the process of scientific research design and data collection. Combining the approaches of the sciences and arts provides opportunities for mutual benefits that are more than the sum of their parts.

Audification and sonification can be used to sense, explore, and understand data, and are essentially the auditory equivalent of data visualisation. Broadly speaking, audification is the most straightforward form of data sonification—transforming data directly into sound—while sonification is a broader term, which refers to conveying information using nonspeech sounds [1]. Our ability as listeners to detect changes in rhythmic patterns, pitch, volume, tempo, timbre, etc. can give insights into changes in scientific model outputs or empirical data. The transformation of data directly into sound has proven its worth in a number of scientifically linked applications, including Geiger counters, which transform ionizing radiation levels into audible clicks [2,3], and pulse oximeters, which emit higher pitches for higher blood oxygen concentrations and are used in medical settings [4]. Audification is particularly suited to situations where we need to have access to a constant stream of data and be able to detect changes in that data while continuing with other activities. This is because the human ear is omnidirectional, detecting sound from all directions continuously, in contrast to eyesight, which can only be used forwards. Additionally, we cannot exclude sound from our ears, unlike shutting our eyes.

The Sonic Kayak project emerged from the Bicrophonic Research Institute (BRI), established by Kaffe Matthews and David Griffiths in 2014 [5]. Through 10 years of international projects the BRI developed the Sonic Bike whose music changes depending on where the cyclist goes. Sound maps are made through residential research and workshop collaborations with producers and communities and then loaded onto the system on the bikes. As the cyclist pedals around the city, a global positioning system (GPS) receiver on the bike detects where they are and triggers site-specific sounds played through a pair of bike-mounted speakers. The Sonic Bike creates an outdoor listening experience for all; the antithesis of headphones—the cyclist becomes a performer and the passers-by, the audience. Politics, time, cycling possibilities, architecture, and finances bear on these new works. Sonic cycling hubs have appeared worldwide, including in Finland, Houston, London, Brussels, and Berlin [5].

The Sonic Kayak project builds directly on the Sonic Bikes. We modified the open-source technology for use on boats and added underwater sensors to take a pure arts project into the realm of citizen science. Underwater sounds, detected by hydrophones (underwater microphones), are played through speakers, along with data from 2 digital thermometers sonified in real time. This allows the paddler to explore the underwater environment through sound. As with the Sonic Bike system, location-specific sounds can also be loaded onto maps and triggered in different places. Meanwhile, the sensor data is recorded with time, date, and GPS location, providing research-quality data of use for marine noise pollution and climate research. The Sonic Kayaks were launched at the 2016 British Science Festival in Swansea, United Kingdom.

In this paper, we outline instructions for building the open-hardware and open-source software for making a Sonic Kayak. We demonstrate the suitability of the sensors used and provide preliminary data from the hydrophones and digital thermometers, considering the potential applications in marine noise pollution and climate research. We discuss the approach of using open hacklabs (events for anyone interested to design and build together) for project development and the use of public events for data collection.

## Building a Sonic Kayak

**Fig 1:**
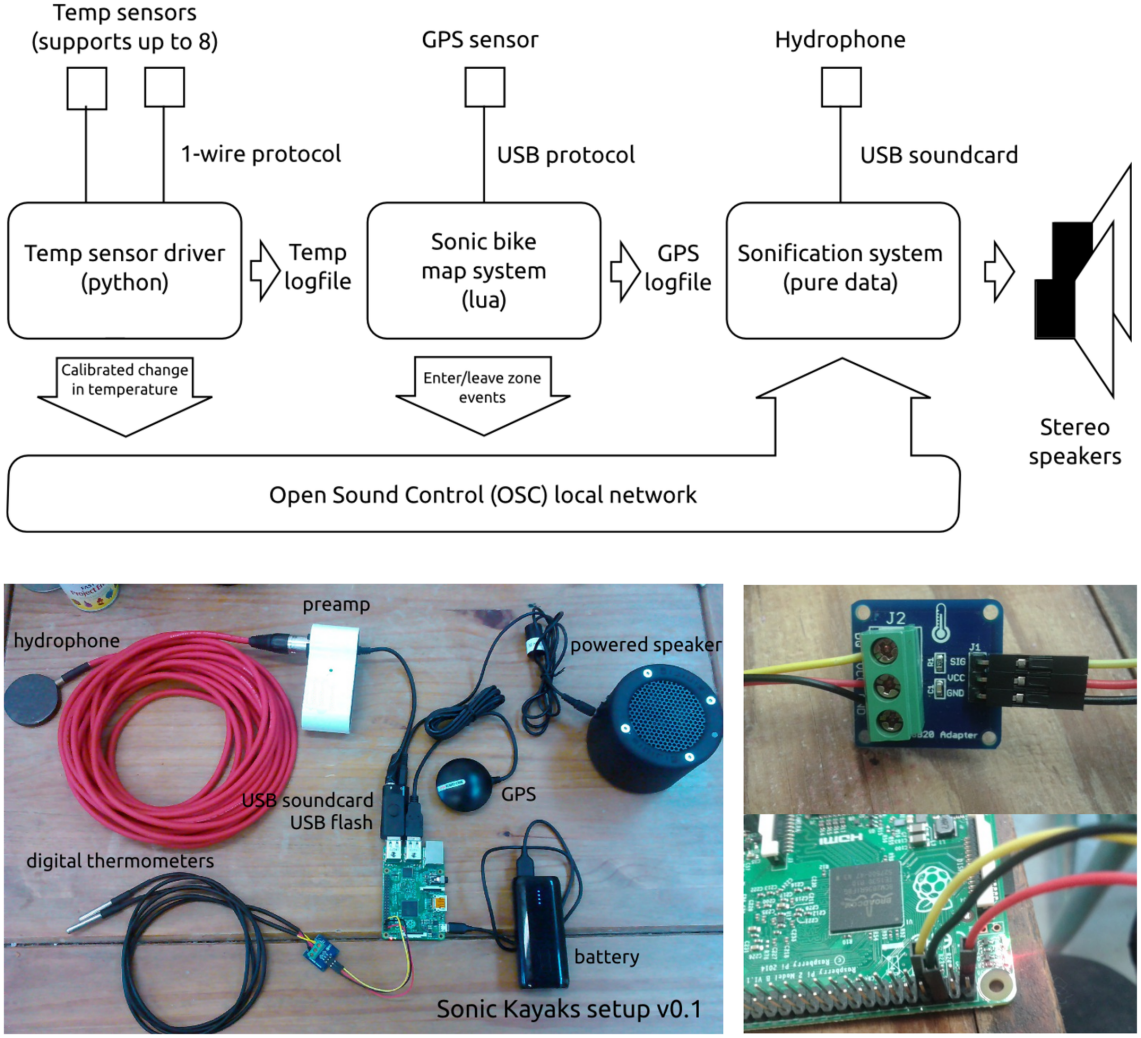
The internal workings of a Sonic Kayak, including a schematic of the software architecture, a photograph of the hardware setup, a close-up of the adaptor between the 1-wire digital thermometer and the Raspberry Pi, and how to connect the adaptor cables to the Raspberry Pi. GPS, global positioning system; temp, temperature; USB, universal serial bus.

The Sonic Kayak system (Fig 1) is run by a Raspberry Pi 2 (£29). This is connected to a universal serial bus (USB) GPS dongle (GlobalSat BU-353-S4 USB GPS receiver, £29), a 5 volt, 3 amp battery (Anker Astro E5 16750mAh portable charger, £30), 2 powered speakers (Minirig, £120 each), a USB soundcard (CSL C-Media USB mini sound card, £10) leading to a pre-amp (Tascam iXZ, plug modified to allow connection to a standard microphone input, £32) leading to a hydrophone (DolphinEar DE-PRO balanced hydrophone, £280), 2 digital thermometers (DX Waterproof DS18B20 temperature sensor with adapter module for Arduino, £3 each, accuracy: ± 0.5°C), and a USB flash drive (Cruzer Glide 16Gb, £5). The temperature sensor was tested for accuracy and response speed against a research-grade marine sensor, and the frequency response of the Raspberry Pi, sound card, and preamp were also tested (full details of these tests are available in S1 Text). The software running on the Sonic Kayaks comprises 3 independent systems (Fig 1; covered in detail in S2 Text) and is available open-source here: https://zenodo.org/record/847727

**Fig 2:**
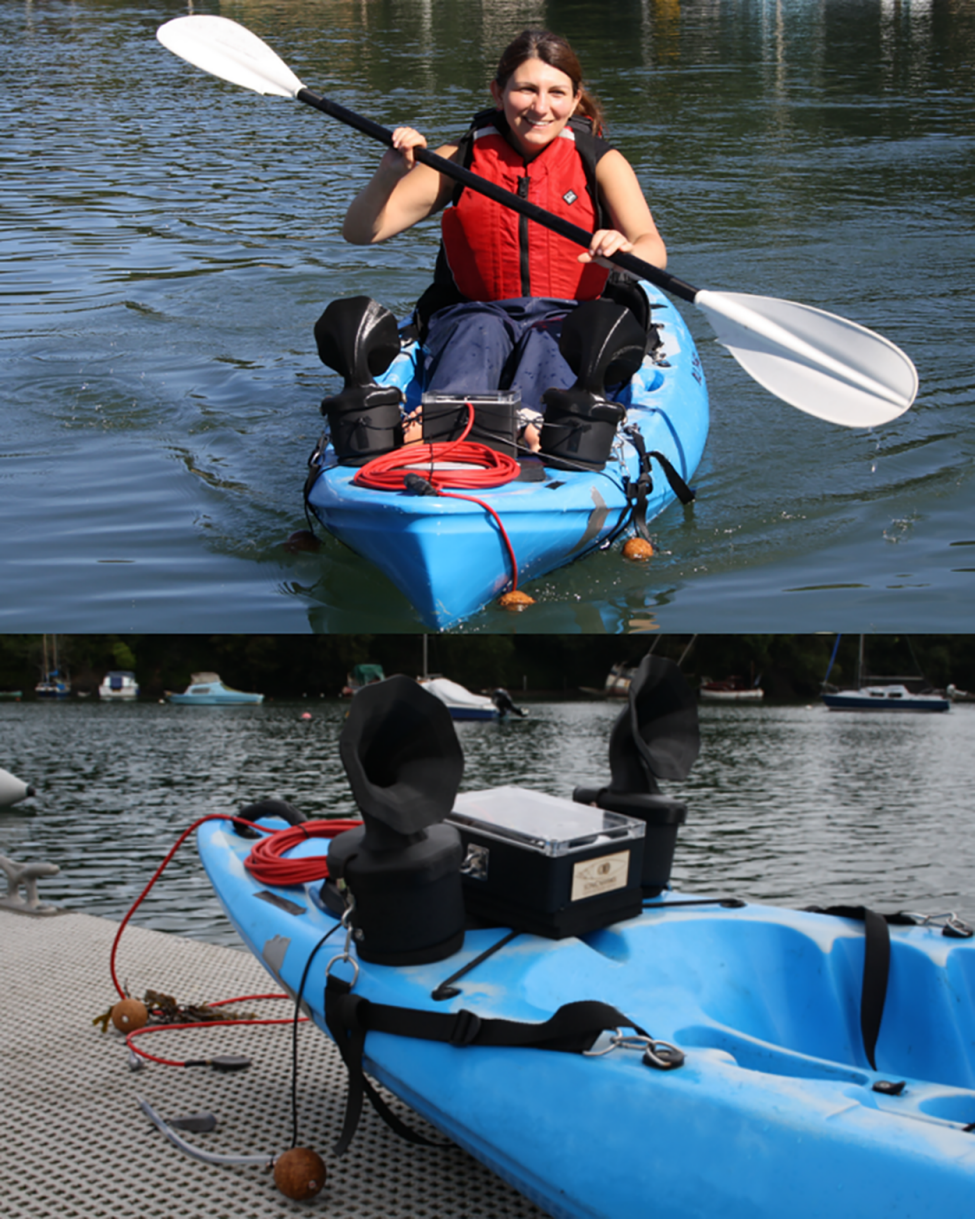
The Sonic Kayak system.

The electronics and speakers were housed in waterproof enclosures and attached to the front of the kayak via custom fixings, allowing the kit to fit to any standard kayak; the materials for waterproofing and fixing cost approximately £60 (S3 Text). We designed 3D-printed, gramophone-style horns to top each of the speakers (shape files available open source here: https://zenodo.org/record/847727, print costs £60 each via Shapeways.com). The complete system (Fig 2) costs approximately £841 in materials, though depending on needs, it would be possible to substantially reduce this cost by omitting or replacing the hydrophone, speakers, and/or printed horns with cheaper versions.

For the sonification, we used 3 layers of sound, including real-time sonification of the data obtained from digital thermometers, a live feed from a hydrophone, and GPS-triggered sounds placed in different geographical locations (S4 Text provides detailed information on the sound design). Recordings from the Sonic Kayaks are available here: http://archive.org/details/sonickayak-recordings. A similar shorter clip was played as part of a 30-minute interview about the Sonic Kayaks on the Cerys Matthews show on BBC Radio 6 Music on August 28, 2016 (available via http://www.bbc.co.uk/programmes/p0464c3r). All samples used are available open source here: http://archive.org/details/sonic-kayak-audio-swansea.

## Citizen collaborations

### Development hacklabs

Instead of developing this project as a small closed team, we launched open invitations for participation and collaboration via two 1-day hacklab events to attract more diverse skills and allow co-creation from the outset and co-learning across disciplines. Participants came with backgrounds in climate change, algal research, fisheries conservation, drone operation, e-textiles, art workshop provision, and computer games (consisting of 11 particpants at the first hacklab and 7 participants at the second hacklab). Prior to the first hacklab, we had not started the development process—embedding the broad perspectives from the start was very beneficial for the project development. During these events, participants were involved in designing and prototyping the system, including working with sound (recording from nature, and sounds generated through instruments and algorithms), developing the audience experience, and testing sensors, coding, and electronics. Much of the software, build design, and sound design used in the final version were developed during these sessions or designed based on feedback from these sessions. We recommend this approach for any citizen science project, or indeed any research project intended to be of use to others.

### Festival event implementation

We built 2 Sonic Kayaks for use in a 2-day installation at the 2016 British Science Festival in Swansea, UK. A video explainer from the British Science Festival event is available here: https://vimeo.com/184935959, which shows the system, how it works, and some of the sounds. The event was fully booked with 64 participants. We ran four, 45-minute sessions each day, with 8 participants per session; within each session there were 2 sonic kayakers and 6 additional kayakers. Each session started with a 10-minute safety briefing, allowing time for the equipment to be checked and adjusted between groups. We worked with the 360 Beach and Watersports Centre on Swansea Bay for the delivery of this event. They provided the kayaks, paddles, and safety gear; gave the safety briefings; and took the groups out each time. For others wanting to run similar events, we strongly recommend that a watersports specialist is actively involved in the project to ensure that basic health and safety issues are met.

We used a pop-up tent and solar panel for storing, fixing, and charging equipment, and provided waterproof books for participants’ comments on their return. The British Science Association used their standard feedback forms for the event, and 21 forms were collected. When rating the event overall, 18 people rated it as ‘excellent’, 2 rated it as ‘good’ and 1 person did not provide a rating (rating response options included the following: excellent, good, average, poor, or terrible). When asked if the event affected their interest in science, 5 people said they were ‘much more interested’, 9 said they were ‘a bit more interested’, 3 said their ‘level of interest was unchanged’, and 3 did not provide a rating (rating response options included the following: ‘I'm much more interested’, ‘I'm a bit more interested’, ‘My level of interest is unchanged’, ‘I'm a bit less interested’, or ‘I'm much less interested’). A total of 17 people provided 3 words to describe the event, and 3 people provided 2 words (available in S5 Text). The most common descriptors were ‘fun’ (as described by 9 people), ‘interesting’ (as described by 4 people), ‘educational’ (as described by 3 people), and ‘thought-provoking’ (as described by 3 people). Feedback gathered from our comment books and talking to participants informally indicated that, after taking the kayaks out, many of the participants understood the climate change aspect to the project despite no specific introduction or discussion of the subject (S5 Text). Two participants were visually impaired and highlighted the possibilities for such a system for auditory explorations of different environments. Some participants had come simply for the opportunity to use a kayak for free. We uploaded the temperature data on an open-source repository immediately following the event. Hydrophone data was not recorded during this event as this feature was developed later.

## Data collection

The digital thermometers and hydrophone tested offer a resolution and replicability that is suitable for research use.

**Fig 3:**
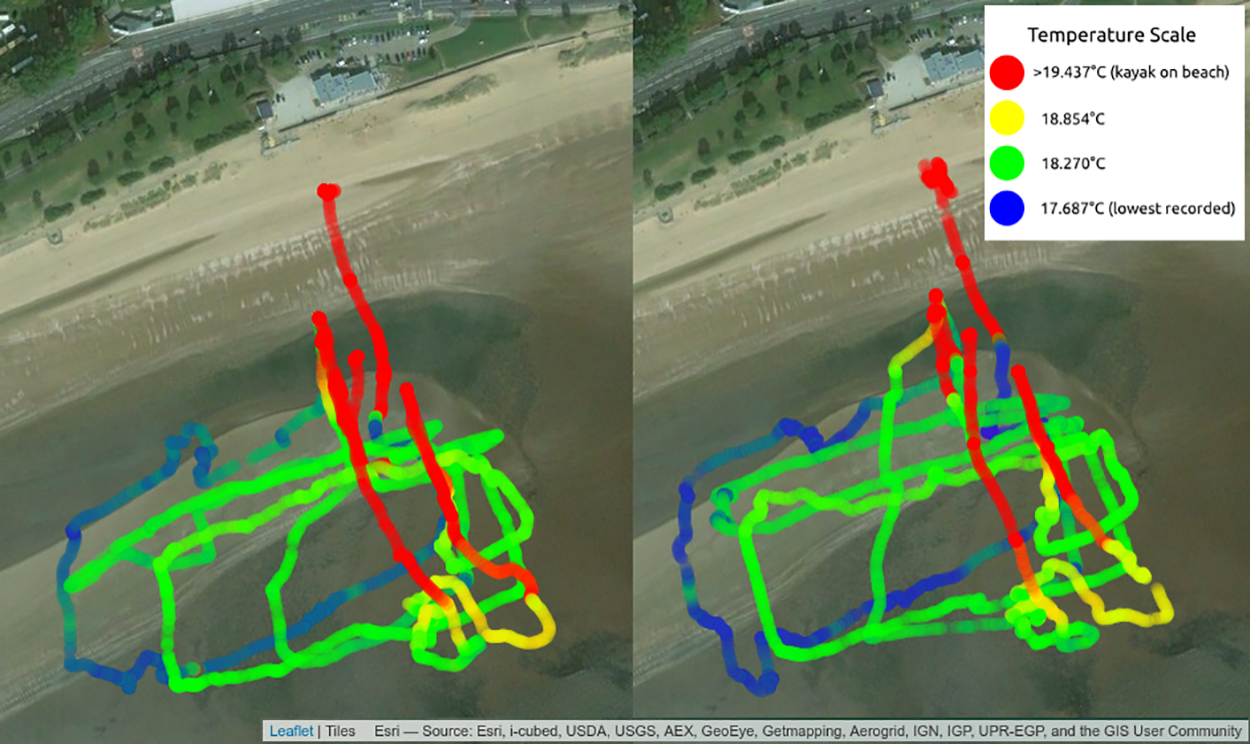
Maps showing the temperature data collected by the 2 Sonic Kayaks at the British Science Festival. Each map shows data from 1 of the kayak systems, with the 4 consecutive trips overlaid. Blue indicates lower temperatures while red indicates higher temperatures. This demonstrates the possibilities for obtaining very fine-scale maps of temperature data over space and time. The temperature information was overlaid with Esri base layer map tiles using the folium leaflet.js python library.

Fig 3 shows the temperature data collected by the festival participants on day 1. Each map is from 1 of the 2 Sonic Kayaks, which took roughly the same routes; the similarity between the data indicates that it is replicable (Kayak 1: minimum temperature = 17.687°C, maximum temperature = 25.687°C, mean temperature = 19.712°C, *n* = 14,431 records; Kayak 2: minimum temperature = 17.812°C, maximum temperature = 23.125°C, mean temperature = 19.792°C, *n* = 13,462 records). The 4 consecutive trips from day 1 have been overlaid on each map. It is possible to see where the boats were taken out of the water between trips (the yellow regions indicate where the temperature rapidly rises) and with successive participant groups as the tide moves out, how the water temperature increases, which shows how the kayaks could be used to detect spatial and temporal microclimatic changes. The raw data is available here: https://zenodo.org/record/847727.

**Fig 4:**
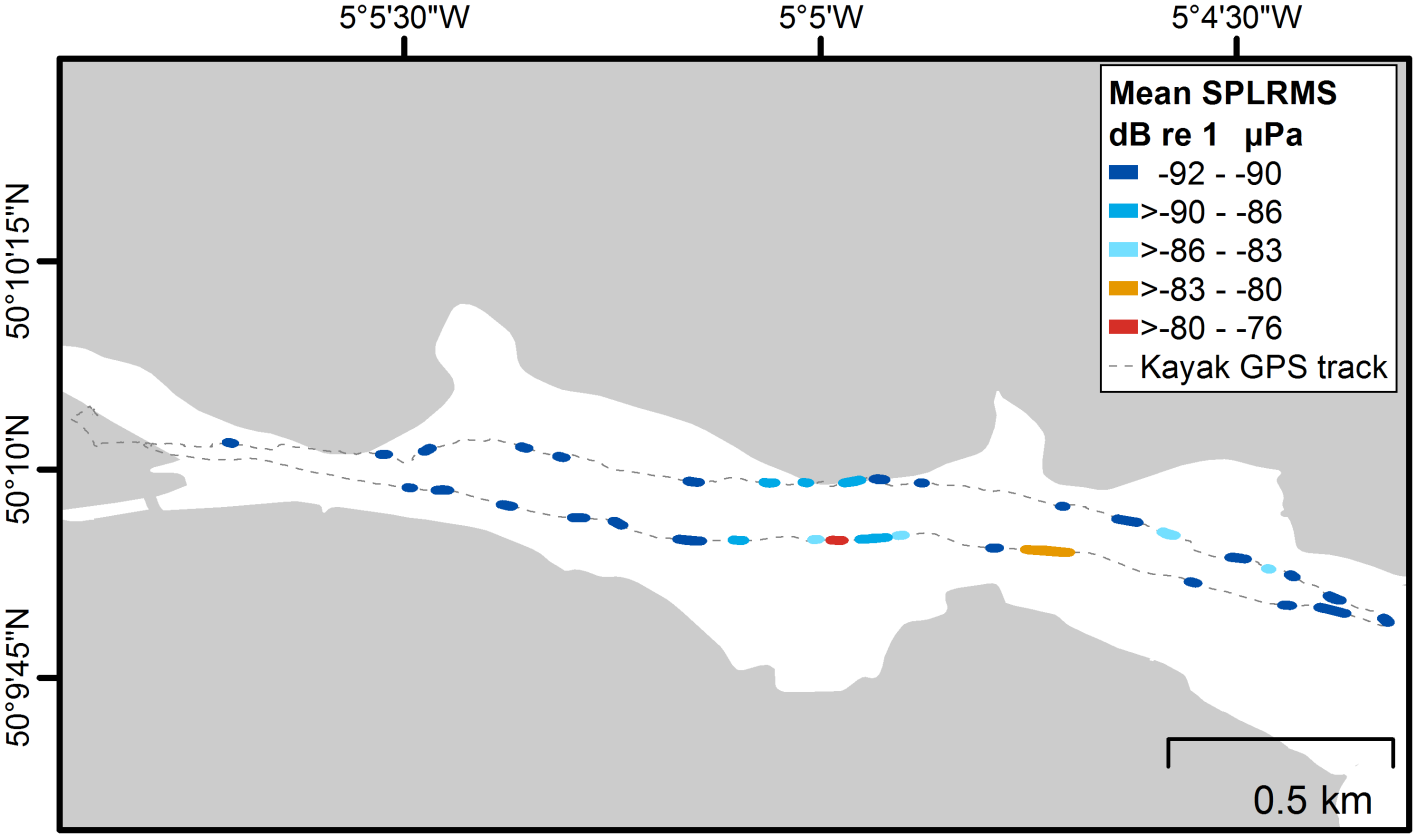
Mean relative SPL_RMS_ dB re 1 μPa (10 Hz–20 kHz) for each section of paused paddling. The sections are displayed with a 10m buffer for ease of visualisation. Figure created in ArcGIS 10.5.1 (ESRI Inc., Redlands, CA). Coastline data from OS Open Map—Local, version 10/2017 (Ordnance Survey, Southampton, United Kingdom). re 1 μPa, referenced to 1 micropascal; GPS, global positioning system SPL_RMS_; root-mean-square sound pressure level.

During testing in an estuary setting, the hydrophone gave insight into man-made noise pollution, particularly from boat motors, noises from wildlife, and the motion of the water. Boat sounds are very loud through the hydrophone; it is possible to hear engines over long distances, which is useful in expanding one’s senses if the boats are out of sight. This is because sound travels 5 times faster underwater than it does in air at approximately 1,500 meters per second, and so it propagates much further. We also heard clicking sounds from underwater species, likely snapping shrimp, chains clinking, background ship noise, flow noise, wind, and waves. The sound levels varied considerably throughout the Sonic Kayak sound tests by 14.6 dB (Fig 4), where we paused kayaking periodically to allow collection of recordings unpolluted by paddling noise. The loudest root-mean-square sound pressure level (SPLRMS), representing the mean broadband sound level for a given time period over a wide frequency range) occurred near to Falmouth Marina and consisted of peaks at specific frequencies from 200 Hz–10 kHz (identified in red in Fig 4, further information can be found in S1 Text).

## Discussion

The Sonic Kayak became a musical instrument, a doorway to listening and looking that creates good health, and a tool with which to collaboratively monitor our changing environment. The launch event at the British Science Festival was fully booked and received good press coverage (including coverage by BBC Radio 6 Music, BBC Radio Wales, BBC World Service, and in local print media), indicating widespread appeal from the combination of sound art, climate, marine science, and sport; the system also has proven potential as a tool for researchers in the fields of underwater noise and climate science.

### Applications for underwater sound research

Underwater noise pollution, such as from commercial shipping, is a potential threat to marine species. A wide variety of effects have been documented including alterations to behaviour, immune responses, and the detection of acoustic signals [6–9]. In extreme cases, anthropogenic underwater noise can directly cause wildlife mortalities [10]. Underwater sound is typically researched by using static sensors fixed to the seabed [11] or by using drifting devices that travel with the current [12]. Sound recordings from towed devices from research boats are also used, for example in cetacean detection [13], but are not generally suitable for the recording of ambient sound due to the noise caused by the motorised vessels themselves. The Sonic Kayak provides the opportunity to generate more fine-scale maps and greater spatial coverage of ambient noise than is possible with static sensors, and provides advantages over drifting recorders by allowing specific sampling locations to be targeted. Kayaks also cause minimal disturbance in comparison to typical research vessels and have the advantage of being able to cover shallow and hard-to-reach locations. The Sonic Kayak system can be used to map both anthropogenic noise pollution and biological noise for the assessment of ecosystem health, potentially enabling better targeted management approaches. Depending on the application of the system, more research may be required to identify and remove the source of electrical noise. Knowledge of the hydrophone sensitivity is also required for the calculation of absolute sound levels, and so this would be essential for comparison with recordings taken using different equipment.

### Applications for climate and ecosystem research

Sea temperatures are typically measured either by using static temperature sensors attached to buoys, ships, or shorelines, or via satellite observations with spatial resolutions of a number of kilometers. Fine-scale spatial and temporal temperature data are not commonly obtained, but are critical for understanding coastal ecosystems, which change over very small distances and throughout the day. The Sonic Kayak’s ability to obtain fine-scale temperature maps, both spatially and temporally, is advantageous for the study of the current and future impacts of climate change on estuarine fish breeding habitats, bettering our understanding of why algal blooms occur in specific areas at certain times [14,15], monitoring the impact of commercial and/or residential outflows or harbours on ecosystem microclimates [16] or the identification of suitable locations for shellfish and fish conservation (e.g., restocking or farming) [17–19], and could equally be applied to river and lake ecosystems as well as coastal. Additionally, the acoustic recording capabilities could be used to monitor the success of resulting conservation interventions. The only modification necessary, depending on the data requirements, might be to fix the depth of the thermometers to ensure that this is kept constant.

### Additional suggested modifications

When the system was trialled in Swansea Bay for the British Science Festival, we experienced fast corrosion from salt water on metal components of the Raspberry Pi and speakers that had voltage differentials; as such, the system would benefit from further waterproofing. The current design is suitable for lake, river, and estuarine use in dry weather, but is not suitable for use where there are waves or in heavy rainfall, nor is it robust enough for long-term implementation. Straightforward options for further waterproofing could include using resin to cover the electronics and hermetically sealing the cable ports.

Additional sensors can easily be added to the existing system, for example instructions for low-cost, open-source turbidity sensors are available and would enable fine-scale monitoring of pollution in estuarine waters from farm runoff [20], and plans for low-cost fluorometry sensors are also available, enabling measurements of phytoplankton fluorescence [21]. The system is fully open source, allowing anyone to make their own version with any required modifications.

The system facilitates citizen-led data collection. The sonification aspect provides feedback to the user, making it more likely that people will enjoy using it and actively seek out the experience; this aspect is frequently not considered, yet it is critical for encouraging uptake and retaining interest. In the future, a live mapping system addition would be beneficial, updating in real time and showing where data gaps are to guide people towards particular locations. In terms of citizen science data collection, GPS-triggered sound zones could bias where users spend time, and so bias the data collected. However, sound zones could also be used as incentives to move to specific areas or as “rewards” for entering an area that few people have gathered data from.

## Conclusions

Testing indicates that the Sonic Kayak system has potential as low-cost research equipment, immediately in the fields of climate change and marine noise, but also in other fields related to water monitoring. The open hacklab approach proved highly valuable, bringing broad perspectives into the development process from the very start, helping to ensure that the Sonic Kayak met its dual purpose both for citizen science and sound art. Combining sound art with citizen science means that paddlers can use sound to explore and investigate the marine environment, and encourages further use of the system as it is an experience in its own right. The sound element also opens unusual opportunities for those with visual impairments, further broadening the range of people who can benefit from such systems. We found that several participants at the British Science Festival had come simply for a chance to use a kayak, highlighting the additional benefit of the sports angle to the project for attracting participants who might otherwise not be interested. It seems fair to conclude that projects which utilise a wide disciplinary approach will be of interest to a broader range of people; this can only be beneficial for citizen science.

## Supporting information

S1 Text Sonic Kayak sensor tests. (ODT)

S2 Text Sonic Kayak software. (ODT)

S3 Text Sonic Kayak fixings. (ODT)

S4 Text Sonic Kayak sound design. (ODT)

S5 Text Sonic Kayak feedback. (ODT)

## Abbreviations

BRI: Bicrophonic Research Institute
GPS: global positioning system
SPL_RMS_: root-mean-square sound pressure level
USB: universal serial bus
USDA: United States Department of Agriculture
USGS: United States Geological Survey
