# Supplementary material for: Sonic Kayaks: Environmental monitoring and experimental music by citizens
Source: PLoS Biol. 2017 Nov 30;15(11):e2004044. doi: 10.1371/journal.pbio.2004044 (PMC5708627; doi:10.1371/journal.pbio.2004044)
Supplement: Supplementary file 1 [file pbio.2004044.s001.odt]

S1 Text. Sonic Kayak Sensor Tests

Temperature Sensor Tests

For the Sonic Kayak system to work, we needed a temperature sensor that could do two things: (i) Gather underwater microclimate data that is high enough quality for scientific research purposes, and (ii) Provide a live-feed of data for sonification purposes. We used a low-cost (£3) waterproof 1-wire digital thermometers (part number[ ](https://datasheets.maximintegrated.com/en/ds/DS18B20.pdf)[DS18B20](https://datasheets.maximintegrated.com/en/ds/DS18B20.pdf)) which was tested against a research-grade marine temperature sensor. The standard temperature sensor for marine research is the TidbiT. These are often attached to marine buoys for long-term temperature data collection. The sensor is small (about the size of a bottle cap), there is a 'shuttle' to connect the sensor to a computer, and proprietary software to programme the sensor before deployment and download/graph the data after deployment. In total this system costs approximately £240. Because TidbiTs can not provide a live data feed, they were not suitable for the Sonic Kayaks, however we used a TidbiT (HOBO TidbiT v2 Water Temperature Data Logger) to check the data obtained from low-cost (£3) waterproof 1-wire digital thermometers (part number[ ](https://datasheets.maximintegrated.com/en/ds/DS18B20.pdf)[DS18B20](https://datasheets.maximintegrated.com/en/ds/DS18B20.pdf)).

A low-cost digital thermometer was wired up to a Raspberry Pi 2 computer (see Fig. SI1-1 showing the adaptor which came with the sensor, and how to wire the adaptor into the Raspberry Pi) and code was written to read/write the sensor data each second (available open source here:[ ](https://github.com/sonicbikes/sonic-kayaks/blob/master/sensors/therm.py)[https://github.com/sonicbikes/sonic-kayaks/blob/master/sensors/therm.py](https://github.com/sonicbikes/sonic-kayaks/blob/master/sensors/therm.py)).

To compare the performance of the sensors, a TidbiT and a low-cost sensor were left in room temperature water, then moved to two flasks at successively higher temperatures for approximately 6 minutes each.

The low-cost digital thermometers monitor the temperature every second, whereas the TidbiT took ~6 minutes to reach the correct temperature - in our tests the low-cost sensor data shows the water in the flasks cooling over time, while the TidbiT is still slowly honing in on the temperature (Fig. SI1-1).

Fig 6.png

Fig SI1-1. Temperature data obtained from a TidbiT sensor (red) and a low-cost digital thermometer (blue), starting in water at room temperature, and moving into two flasks of water at successively higher temperatures for ~6 minutes each.

The datasheets indicate that the low-cost sensors are slightly less accurate than the TidbiT (TidbiT Range: -20 to 70°C, Accuracy: ± 0.21°C, Resolution: 0.02°C, Response time: 5 mins; Low-cost 1-wire digital thermometer Range: -55 to 125°C, Accuracy: ± 0.5°C, Resolution: 0.063°C, Response time: not stated), however our results indicate that the low-cost sensors provide better data resolution due to their faster response time, with an accuracy level equivalent to the TidbiT.

Hydrophone Tests

Audio from the DolphinEar DE-PRO Balanced Hydrophone was recorded via Pure Data onto a USB flash drive (16 bit recording, 0 dB gain, single .wav audio file per kayak trip). This hydrophone has a recording bandwidth of 10 Hz - 24 kHz with an approximately flat (±2 dB) response 20 Hz - 20 kHz (-6 dB at 10 Hz and 24 kHz).

All acoustic data processing was carried out in MATLAB (2016b, The Mathworks). Tones at frequencies 10 Hz, 60 Hz, 200 Hz, 1000 Hz (1 kHz), 10000 Hz (10 kHz) and 20000 Hz (20 kHz), with known voltages as found using an oscilloscope, were recorded through the entire system (Raspberry Pi, pre-amp and sound card). The recorded voltage was then divided by the input voltage for each frequency to calculate the correction factor required. The values were interpolated using a standard linear interpolation method in MATLAB to provide a correction factor per 1 Hz.

During initial tests, paddling noise was pervasive - therefore for the purposes of scientific data collection the sampling procedure was to collect unpolluted samples by pausing paddling. Test trips were made with two Sonic Kayak systems on the Penryn estuary in Cornwall in March 2017 on an incoming tide. Pausing paddling to collect unpolluted recordings was limited by the windy conditions at the time. As well as pausing periodically where possible we also paused when sounds of interest were heard through the onboard speakers, for example, boats.

The hydrophone sensitivity is not available from the supplier, therefore only relative dB levels are calculated. To calculate the sound levels per 1 Hz a Fast Fourier Transform (FFT) was applied sequentially to overlapping 1 second segments (Hann window, 50% overlap). This process converts the waveform signal in the time domain to the frequency domain. The calculated correction factor is applied during this process. The results are converted into dB using the equation:

formula

where pRMS is the result from the FFT process as root-mean square pressure, and pref is the reference pressure (the decibel scale is a logarithmic ratio scale). In underwater acoustics, the reference pressure is 1 µPa which is different to the reference pressure used for sound in air and they are therefore not directly comparable.

The root-mean-square Sound Pressure Level (SPLRMS) represents the mean broadband sound level for a given time period over a wide frequency range. Mean SPLRMS (1 s window, 50% overlap; 10 Hz - 20 kHz) were calculated for each period of paused paddling. For each 1 s window, an FFT process was first applied and the frequency-dependent correction factors applied. The result was converted back into the waveform and the SPL was calculated using the equation:

formula

where pRMS is the root-mean-square pressure, padj is the corrected instantaneous pressure and t is the time (in number of datapoints). The mean SPLRMS was calculated for each period of paused paddling and then converted into dB using the same equation as above.

Figure SI1-2 shows the path taken during the hydrophone tests, as monitored by the on-kayak GPS. Self-noise was present at several frequencies with peaks at 3,798 Hz, 4,138 Hz, 7,925 Hz, 8,679 Hz, 11,712 Hz, 12,809 Hz, and 20,679 Hz - this is thought to originate from the Raspberry Pi or the GPS after the elimination of all other components. Paddling produced a broad peak ~300 Hz and peaks at 500 Hz and ~2 kHz (Fig. SI1-3). When paddling, the noise masks other sounds at similar frequencies.

## Fig SI1-2. Map showing the Sonic Kayak test GPS track, March 2017. Figure created in ArcGIS 10.5.1 (ESRI Inc.). Coastal data from OS Open Map – Local, version 10/2017.

Fig 8.jpg

Fig SI1-3. a) Mean power spectral density (relative dB) of the paddling noise given in (b), giving the average relative sound levels per 1 Hz for the entire duration presented in (b).  b) Spectrogram of a sample of paddling noise displaying the sound levels per 1 Hz per 1 s (50% overlap; time interval is 0.5 s).

Periodically and when sounds of interest were heard through the speakers, paddling was paused to record unpolluted samples. For each of these samples, the mean broadband sound level was calculated for the frequency range 10 Hz - 20 kHz (SPLRMS re 1 µPa). These levels varied considerably throughout the Sonic Kayak sound test by 14.6 dB (Fig. 9). The loudest SPLRMS (identified in red in Fig. 3 in the main text and repeated below as Fig SI1-4) occurred near to Falmouth Marina and consisted of peaks at specific frequencies from 200 Hz - 10 kHz (Fig. SI1-5).

Fig 9.png

Fig SI1-4. Mean relative SPLRMS dB re 1 µPa (10 Hz - 20 kHz) for each section of paused paddling. The sections are displayed with a 10 m buffer for ease of visualisation.

Fig 10.jpg

Fig SI1-5. Mean power spectral density (relative dB) of the loudest mean SPLRMS identified giving the average relative sound levels per 1 Hz for the entire duration presented in (b). b) Spectrogram of the loudest mean SPLRMS identified displaying the sound levels per 1 Hz per 1 s (50% overlap; time interval is 0.5 s).
