# Supplementary material for: Sonic Kayaks: Environmental monitoring and experimental music by citizens
Source: PLoS Biol. 2017 Nov 30;15(11):e2004044. doi: 10.1371/journal.pbio.2004044 (PMC5708627; doi:10.1371/journal.pbio.2004044)
Supplement: Supplementary file 2 [file pbio.2004044.s002.odt]

S2 Text. Sonic Kayak Software

The software is built from three independent systems, one is written in Lua and is based on the original Sonic Bike system. This is responsible for reading the GPS device and creating events for when the kayak enters or leaves a GPS zone defined on a supplied map. This system also records the GPS location in a logfile every second. The temperature sensor driver is written in Python and reads the two digital thermometers using the 1-wire serial protocol on the Raspberry Pi General Purpose Input/Output (GPIO) port, logs the reading and creates a secondary calibrated value to give highly sensitive indications of rises or falls in temperature. These readings, along with the enter/leave zone events are passed to the sonification system via Open Sound Control messages (OSC). The sonification system is written in Pure Data, an open source visual programming language. Pure Data converts the thermometer data into sound - via sample playback in the case of zone events, or synthesising the sound directly from the data in the case of temperature changes. The sonification system also reads the hydrophone sound, amplifies it and passes it directly to the output - which is fed to the two speakers. Figure 1 provides a schematic of the different systems and how they feed into each other. The software running on the Sonic Kayaks is available open-source here:[ ](https://github.com/sonicbikes/sonic-kayaks)[https://github.com/sonicbikes/sonic-kayaks](https://github.com/sonicbikes/sonic-kayaks)
