# Supplementary material for: Sonic Kayaks: Environmental monitoring and experimental music by citizens
Source: PLoS Biol. 2017 Nov 30;15(11):e2004044. doi: 10.1371/journal.pbio.2004044 (PMC5708627; doi:10.1371/journal.pbio.2004044)
Supplement: Supplementary file 3 [file pbio.2004044.s003.odt]

S3 Text. Sonic Kayak Fixings

The Raspberry Pi, GPS, battery and preamp were housed in a waterproof box, while the speakers were housed in customised cylindrical tupperware tubs. The box and speaker tubs were joined with flexible metal links (screw-in D-loops and metal clasps, easily found in any chandlery or good DIY shop). The box and speaker tubs were placed on top of the kayak at the front, and attached to the kayak using custom designed adjustable nylon webbing straps, running under the bottom on the kayak and attaching to the handles on either side of the kayak. The hydrophone was placed off the front end of the kayak (secured with cable ties) with a weight close to the sensor and a float approximately 1m along the cable to keep the sensor at a depth of 1m. The digital thermometers were placed over each side of the kayak, with a weight close to the sensor and a float approximately 60cm along the cable. Neoprene pads (1cm thick) were cut to fit under the box and speaker tubs to ensure a tight fit to different kayak models. In total, the material for fixing and waterproofing cost approximately £60. We designed and 3D printed gramophone-style horns to top each of the speakers (shape files available open-source here:[ ](https://github.com/sonicbikes/sonic-kayaks/blob/master/hardware/horn-print.stl)[https://github.com/sonicbikes/sonic-kayaks/blob/master/hardware/horn-print.stl](https://github.com/sonicbikes/sonic-kayaks/blob/master/hardware/horn-print.stl), print costs £60 each via Shapeways.com). 

The electronics and housing design worked well throughout four hours of continual use on the first day of the British Science Festival. However, we did notice some corrosion from salt water on metal components of the Raspberry Pi and speakers that had voltage differentials. In addition, towards the end of the four hours, there was condensation inside the main electronics box, caused by the components heating up inside, surrounded by a colder environment outside the box. On the second day, both kayaks were capsized for several minutes due to difficult waves, and the session was called off. Remarkably, one set of Sonic Kayak hardware remained fully functional with the exception of one speaker - for the other set the waterlogging was terminal.
