# Supplementary material for: Sonic Kayaks: Environmental monitoring and experimental music by citizens
Source: PLoS Biol. 2017 Nov 30;15(11):e2004044. doi: 10.1371/journal.pbio.2004044 (PMC5708627; doi:10.1371/journal.pbio.2004044)
Supplement: Supplementary file 4 [file pbio.2004044.s004.odt]

S4 Text. Sonic Kayak Sound Design

We used three layers of sound on the Sonic Kayaks - real-time sonification of the data obtained from digital thermometers, a live-feed from a hydrophone, and GPS-triggered sounds placed in different geographical locations. Sound was played through on-board speakers - the use of headphones should be avoided for safety reasons (being closed off from surrounding above-water sounds is not desirable when on a boat, there is a possibility of tangling in cables if using wired headphones, and a risk of electric shock if they get wet). Below each layer of sound is addressed in turn.

Digital Thermometer Sonifications

Data from the two digital thermometers on each boat were sonified in real-time. As the water temperature goes up the sound pitch goes up, and vice-versa, with no sound when the temperature is stable. This was designed to provide easy-to-understand sonification of the underwater temperatures while not producing unnecessary noise that could detract from the peaceful nature of kayaking. The pitch is normalised to the running minimum and maximum the kayak has observed. This makes it much more sensitive, but it takes a few minutes to self calibrate at the start. Currently the pitch ranges from 70 to 970 Hz, with slight frequency modulation at 90 Hz to make the lower end more audible.

Originally we designed the temperature sonification to be continuous. It proved important that no sound was produced when the temperature was stable as during testing, users remarked on wanting more quiet time while out on the water. This also served to keep kayakers aware of their above-water surroundings, which is advantageous from a safety perspective.

Hydrophones

A hydrophone was used to transduce live underwater sounds continuously. Even with a preamp the hydrophone needed to be boosted in Pure Data by 12 times to allow the paddler to hear detailed sounds. During testing in an estuary setting, the hydrophone gave insight into man-made noise pollution particularly from boat motors, and noises from wildlife and the motion of the water. Boat sounds are very loud through the hydrophone - it is possible to hear engines over long distances, which is useful in expanding your senses if the boats are out of sight. This is because sound travels five times faster underwater than it does in air at ~1500 ms-1, and so propagates much further. We also heard clicking sounds from underwater species, likely snapping shrimp, chains clinking, background ship noise, flow noise, wind and waves. In Swansea Bay the environment was much more homogenous underwater (sand) and noisy above water (waves) – the hydrophone contributed very little in this setting, so having the additional GPS triggered sounds proved an important addition. While testing in the relatively quiet estuarine setting, the system could clearly be heard from onlookers on land - in this case, as with the Sonic Bike system that came before, the paddlers inherently become performers and passers-by become an audience.

GPS-Triggered Sound Zones

Geographical locations were mapped with sounds attributed to them, using custom software. The GPS on the kayaks detected when the boat was within one of these zones, triggering the sounds to play. During testing it became apparent that there were divergent views on this additional layer of sound. Some users preferred more quiet and only to have the temperature sonification and hydrophone sounds, while others wanted additional sounds to make the experience more interesting. The final design for the British Science Festival implemented bands of sound allowing the paddler to choose their experience - we mapped three bands of sound running out from the shore, to allow for the tide changing dramatically over the course of the day (Swansea Bay has the longest tidal range in Europe). One zone contained poetry about climate change and the sea, another zone contained beats and music, and the third zone contained electronic voices with factual messages about climate change. Between each zone there were areas where no additional sounds were present, allowing the paddler to choose how they wanted to use the kayaks.
