# Supplementary material for: Sonic Kayaks: Environmental monitoring and experimental music by citizens
Source: PLoS Biol. 2017 Nov 30;15(11):e2004044. doi: 10.1371/journal.pbio.2004044 (PMC5708627; doi:10.1371/journal.pbio.2004044)
Supplement: Supplementary file 5 [file pbio.2004044.s005.odt]

S5 Text. Sonic Kayak Feedback

Free words to describe the Sonic Kayak event as provided by festival participants using British Science Association standard feedback form:

Free comments about the Sonic Kayak event as written in the comments books made available at the British Science Festival:
